# RNA circularization strategies *in vivo* and *in vitro*

**DOI:** 10.1093/nar/gkv045

**Published:** 2015-02-06

**Authors:** Sonja Petkovic, Sabine Müller

**Affiliations:** Institut für Biochemie, Ernst Moritz Arndt Universität Greifswald, Felix-Hausdorff-Str. 4, 17487 Greifswald, Germany

## Abstract

In the plenitude of naturally occurring RNAs, circular RNAs (circRNAs) and their biological role were underestimated for years. However, circRNAs are ubiquitous in all domains of life, including eukaryotes, archaea, bacteria and viruses, where they can fulfill diverse biological functions. Some of those functions, as for example playing a role in the life cycle of viral and viroid genomes or in the maturation of tRNA genes, have been elucidated; other putative functions still remain elusive. Due to the resistance to exonucleases, circRNAs are promising tools for *in vivo* application as aptamers, *trans*-cleaving ribozymes or siRNAs. How are circRNAs generated *in vivo* and what approaches do exist to produce ring-shaped RNAs *in vitro*? In this review we illustrate the occurrence and mechanisms of RNA circularization *in vivo*, survey methods for the generation of circRNA *in vitro* and provide appropriate protocols.

## INTRODUCTION

Circular RNAs are found in all kingdoms of life, appearing for example as genomes of viroidal plant pathogens (1) and of the *hepatitis delta* virus (HDV) (2), or as spliced tRNA and rRNA introns and as rRNA processing intermediates in archaea (3,4). Furthermore, circRNAs are formed in the life cycle of bacterial and eukaryal group I introns, where they were suggested to play a role in intron mobility by reverse splicing (5–7). However, circRNAs were considered extremely rare in nature for decades, and in particular in eukaryotes, circRNAs were seen as minor RNA structural variants attributed to transcriptional noise (8). This view has dramatically changed, as a number of recent reports have convincingly demonstrated that circRNAs in eukaryotes are highly abundant and evolutionary conserved. Apparently, thousands of human transcripts are expressed as circular isoforms of their linear counterparts (9–13). The functions of circRNAs in eukaryotes still remain more or less elusive, although it has been suggested that circRNAs may act as transcription regulators (10) or as competing endogenous RNAs to bind miRNAs (RNA sponges) or RNA binding proteins (protein sponges), and thus to modulate their local free concentration (11–15). Moreover, it was suggested that circRNAs might encode proteins with functions distinct from those of their canonical linear counterparts. At least *in vitro* translation of circRNAs was demonstrated (16). Very interestingly, circRNAs were described as biomarkers of aging in Drosophila (17) and as putative disease biomarkers in human saliva (18). However, the large number of detected circRNAs is also critically discussed. There is some agreement that artifacts of RT-PCR detection may add to the large number of circRNAs. A recent report raises serious doubts regarding the biological function of most circRNAs. Based on the results of a computational approach for identification and analysis of circRNAs, the authors suggest that apart from CDR1as, which indeed appears to function as miR-7 sponge (11,12), a large majority of circRNAs might be just inconsequential side products of pre-mRNA splicing (13). Nevertheless, as mentioned above, there is also good agreement that circRNAs may fulfill important functions, although not much is known yet.

What might be the advantage of RNA being in a circular form rather than in the traditional linear one? The most obvious reason is certainly stability. Since exonucleases are ubiquitously spread, circRNAs are advantageous in terms of being protected against degradation. The covalently closed ring structure may be not only favorable for endogenous RNAs, but should also be beneficial to the application of RNAs for example as antisense-RNAs, aptamers, ribozymes or siRNAs (19–23).

What are the strategies for production of circRNAs? *In vivo*, circRNAs usually result from splicing events, either as exonic circRNA from circularization of exons or as intronic circRNA, such as, for example, circular tRNA and rRNA introns produced from archaeal splicing. Circular RNAs appear in viruses and viroids and as viroid-like satellite RNAs. *In vitro*, RNA circularization involves the intramolecular formation of a 3′, 5′-phosphodiester bond, requiring close proximity of the 3′- and 5′-terminus of the linear precursor. Here we review pathways of circRNA formation *in vivo* and strategies for RNA circularization *in vitro*. Furthermore, we provide a collection of protocols to be used for the purpose of RNA circularization (Supplementary data).

## CIRCULARIZATION *IN VIVO*

### Formation of exonic circRNAs

**Figure 1.**
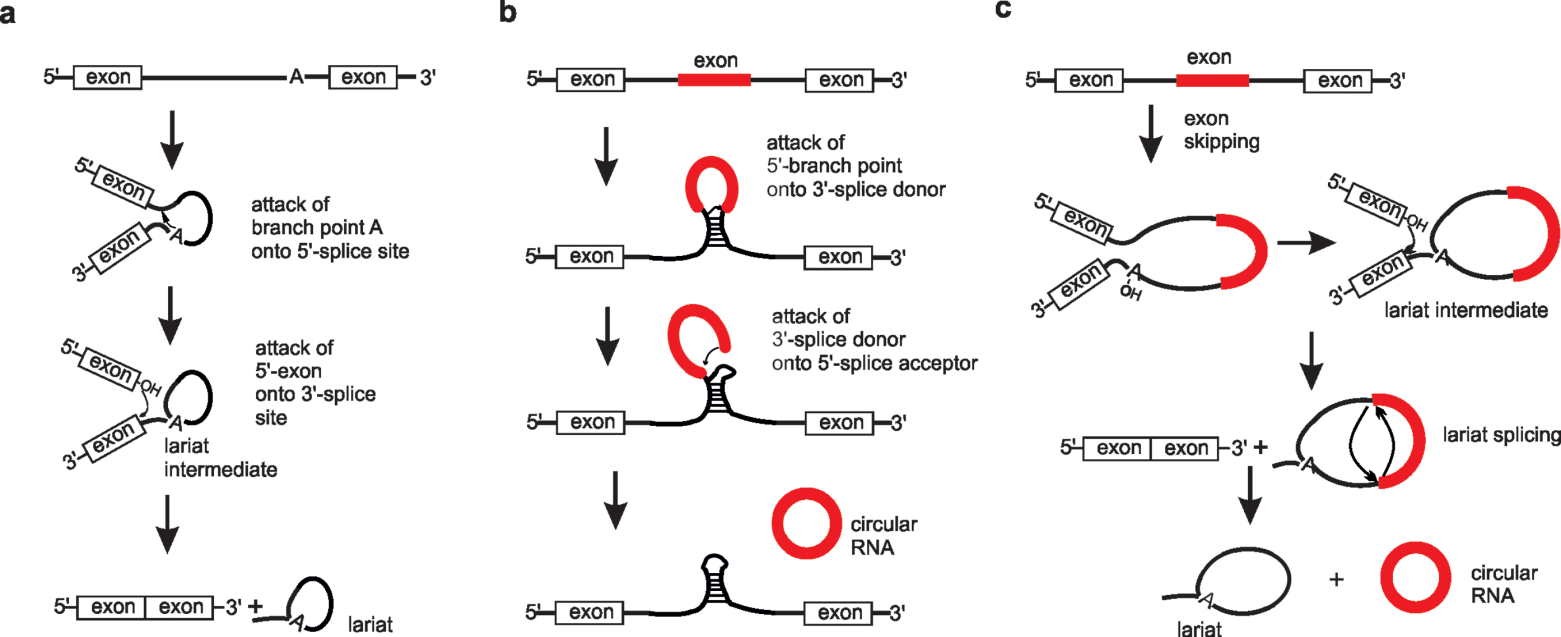
Regular linear splicing (**a**) and two models for the formation of exonic circRNAs (**b, c**). (a) Upon folding, the branch point adenosine (bpA) attacks the 5′- splice site, delivering the 5′-exon with free 3′-OH group and the lariat intermediate with the intron still linked to the 3′-exon. Nucleophilic attack of the 3′-OH group of the 5′-exon onto the 3′-splice site leads to ligation of the two exons, and to release of the intron as lariat. (b) Direct backsplicing. Two unspliced introns interact by complementary base pairing, thereby juxtaposing the branch point of the 5′-intron and the 3′-intron–exon junction (3′-splice donor) for nucleophilic attack and cleavage. Then, the 3′-splice donor attacks the 5′-intron–exon junction (5′-splice acceptor) joining the two introns and releasing the circularized exon. (c) Exon skipping. Through skipping of an exon, an exon containing lariat is created following the normal mechanism of splicing. Backsplicing then occurs as described above, but within the lariat. As a result, the intron lariat is released and a circular RNA is produced.

In eukaryotic cells the spliceosome acts to remove introns from primary transcripts in a two-step mechanism. In the first step, the 2′-OH group of a defined adenosine within the intron (branch point adenosine) attacks the 5′-splice site, generating a free 3′-OH group at the 5′-exon and the lariat intermediate. The second step involves nucleophilic attack of the generated 3′-OH group onto the 3′-splice site, producing the final products: an excised lariat intron and a linear RNA composed of the two combined exons (Figure 1a).

In addition, exonic circRNAs may result from spliceosomal action. They were first observed in 1991 (24). Since then, thousands of endogenous circRNAs have been identified in mammalian cells, some of them highly abundant and evolutionary conserved (for recent reviews see (25–27)). The detailed mechanism of circRNA biogenesis has remained elusive. Currently, two major mechanisms involving the canonical spliceosome are discussed: (i) direct backsplicing and (ii) exon skipping (Figure 1b and c).

What is referred to as ‘direct backsplicing’ (25) was historically termed ‘mis-splicing’ by exon shuffling or exon scrambling, where exons are spliced in non-canonical order (8). However, taking into account that circRNAs may be generated by purpose rather than resulting from mis-splicing events, ‘backsplicing’ is a more appropriate name. Mechanistically, ‘direct backsplicing’ involves joining of the 3′-tail of an expected downstream exon to the 5′-head of an exon that is normally upstream. The downstream splice donor pairs with an unspliced upstream splice acceptor. As a result, the exon becomes circularized (8) (Figure 1b). The second mechanism involves creation of a lariat containing an exon produced from exon skipping. This lariat subsequently undergoes internal splicing, thereby removing the intron and generating a circRNA (28,29) (Figure 1c). Both mechanisms are plausible *in vivo*, although direct backsplicing is favored as the more frequently used pathway (25). In addition, it cannot be ruled out that multiple mechanisms are involved in exonic circRNA formation. Recent findings indicate that exon circularization is dependent on flanking intronic sequences (30–34). RNA–RNA interactions across flanking introns compete with pairing within individual introns and thus determine the efficiency of exon circularization (30–34). Furthermore, it was demonstrated that circularization and linear splicing compete against each other, assigning circRNAs a functional role in gene regulation (15,34).

In order to produce a desired circRNA *in vivo*, overexpression vectors have been designed which include the exon to be circularized and partial sequences of the flanking introns to produce pairing, but missing additional upstream and downstream exonic sequences. Upon delivery in mammalian cells, these vectors were shown to successfully produce circRNAs (11,12).

### Formation of intronic circRNAs

**Figure 2.**
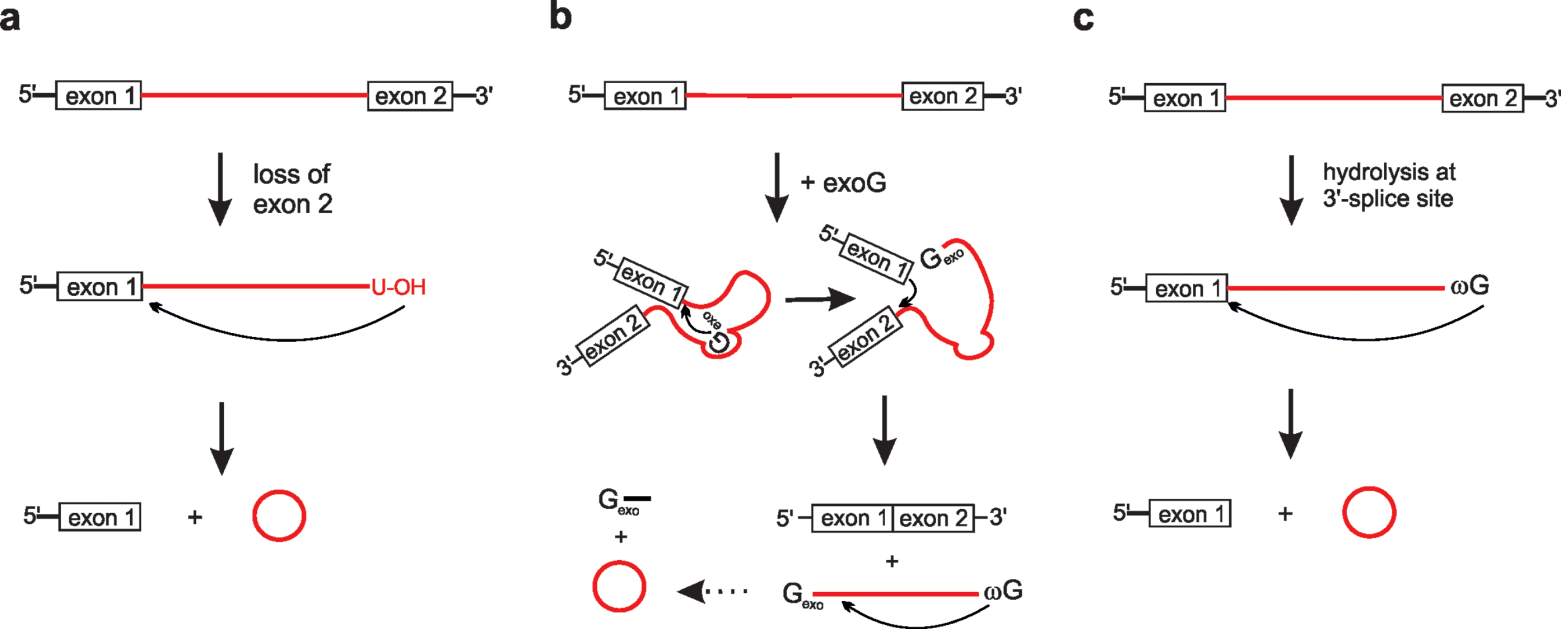
Formation of intronic circRNAs. (**a**) Group II intron mediated circRNA formation. Circle formation requires prior release of the 3′-exon. The terminal 2′-OH group of the intron attacks the 5′-splice site, creating a circular RNA by 2′,5′-phosphodiester formation. (**b**) Group I intron supported regular splicing. An exogenous guanosine (exoG) bound in the intron structure serves as nucleophile attacking the 5′-splice site. Upon first transesterification, the 5′-exon is cut off and exoG becomes linked to the intron. The terminal 3′-OH group of the 5′-exon then attacks the 3′-splice site, the ligated exons and a linear intron are released. Eventually the linear intron is circularized by nucleophilic attack of 2′-OH group of the terminal guanosine (ωG) onto a phosphodiester bond close to the 3′-end and release of a short 3′-tail. Note that in this case a 2′-5′-phosphodiester bridge closes the circle. (**c**) Prior hydrolysis of exon 2 allows circle formation by direct nucleophilic attack of ωG onto the 5′-splice site.

Group II self-splicing introns generate a branched lariat-intermediate and a lariat-intron using the same chemistry as the spliceosome described above (Figure 1a). In addition, there is also evidence for a mechanism, where group II introns are excised as RNA circles, although circularization occurs by formation of a 2′, 5′-phosphodiester bond (35,36). Circle formation requires prior release of the 3′-exon, for example by a *trans*-splicing mechanism. The terminal 2′-OH group of the intron attacks the 5′-exon–intron junction (5′-splice site), thus generating the circularized intron and the 5′-exon (Figure 2a).

In contrast to the spliceosome and group II introns, group I introns self-splice by first recruiting guanosine (exoG) as an external nucleophile that initiates splicing by nucleophilic attack onto the 5′-splice site and thereby becomes attached to the 5′-end of the intron. Upon second transesterification, exons are ligated, and a linear catalytic intron is released (reviewed in (37)). Interestingly, the excised linear intron can undergo circularization by nucleophilic attack of the 3′-terminal guanosine onto a phosphodiester bond near the 5′-end of the intron (38). The 5′-terminal sequence is released and the intron is circularized (Figure 2b). The choice of the circularization site depends on pairing of the three nucleotides preceding the cleaved phosphate to a specific binding site within the intron, thus defining the phosphate to be attacked. As a result, a variety of truncated intron circles are formed, which, however, appear to be short-lived *in vivo* (39).

In addition to truncated circles also formation of full length intron circles was observed. This pathway is initiated by hydrolytic cleavage at the 3′-splice site followed by nucleophilic attack of ωG onto the 5′-splice site (40). The final products are a circular full-length intron and non-ligated exons (Figure 2c). For the *Tetrahymena* intron, full-length intron circles were reported to be minor and barely detectable *in vivo* (40). However, for more complex nuclear group I introns like the one from *Didymium iridis*, full-length circular introns are formed as major product at splicing conditions *in vitro* (41,42), and are also easily detectable *in vivo* (43). Here, truncated circular introns were not observed. The ability of forming full-length intron circles seems to be a general feature of all types of nuclear group I introns. The function of truncated and full-length circular introns is not known, but it was hypothesized that these structures could play a role in intron mobility (5).

### Formation of circular RNAs in archaea

In archaea, circRNAs were mainly observed in tRNA and rRNA introns and in rRNA processing intermediates. Archaeal introns are cleaved from precursor-RNAs by the assistance of a protein, the archaeal splicing endonuclease, and are subsequently ligated by an RNA ligase to form circRNA (3,4). The splicing endonuclease recognizes a bulge-helix-bulge motif, composed of a 4-base pair stem flanked by two 3-nucleotide bulges (44–46). Cleavage occurs at a specific site in the bulge regions, generating characteristic fragments with terminal 5′-hydroxyl group and 2′, 3′-cyclic phosphate (47). Circularization proceeds by nucleophilic attack of the 5′-OH group onto the 2′, 3′-cyclic phosphate of the same molecule forming a 3′, 5′-phosphodiester bridge (3,4). In addition to tRNA and rRNA introns, archaeal 16S and 23S rRNAs were found to be excised as circular intermediates during rRNA maturation. Both are spliced out of a single RNA precursor before being further processed to the mature rRNAs (48). Archaeal circular RNAs presumably have diverse biological functions, many of them yet unidentified. A recent transcriptome-wide search for circular RNAs in archaea discovered, in addition to excised tRNA and rRNA introns, a number of enriched circular non-coding RNAs, including C/D box RNAs and RNase P (49).

### Circular RNAs in viroids, viroid-like satellite RNAs and in the HDV

**Figure 3.**
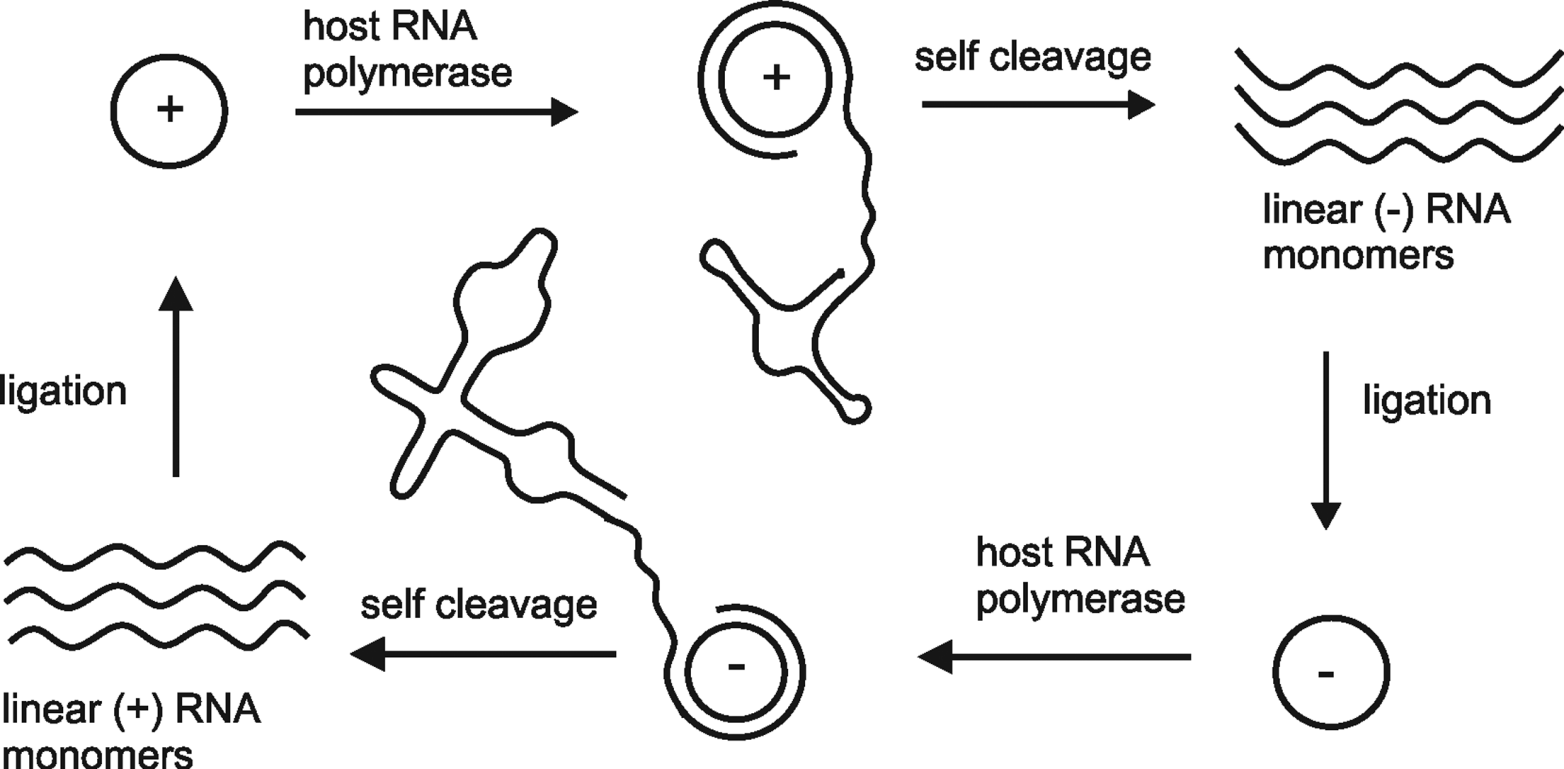
Double rolling circle mechanism as occurring in viroid-like satellite RNAs (50). Here, self-cleavage of the multimeric (−)-strand is mediated by a hammerhead ribozyme, whereas self-cleavage of the multimeric (+)-strand occurs by hairpin ribozyme action. A similar mechanism is employed in the hepatitis-δ-virus replication, as well as in other satellite RNAs and viroids.

Viroid genomes, viroid-like satellite RNAs and the human HDV genome are circular RNAs. They replicate through a rolling-circle mechanism, using the circular template of one or both polarities for reiterative transcription, followed by cleavage of the produced oligomeric RNA into monomeric species and ligation to a circular RNA (1,50–51) (Figure 3). In some species, cleavage is mediated by host enzymes, while in others *cis*-acting ribozymes (hammerhead ribozyme (HHR), hairpin ribozyme (HPR), HDV ribozyme) perform this reaction. Intramolecular ligation appears to be catalyzed mainly by host enzymes, although *in vitro* self-ligation was also observed (52). Mechanistically, there is some variety among the different members of viroid families, satellite RNAs and the HDV virus. Cleavage produces 5′-phosphate and 3′-OH termini or 5′-OH and 2′, 3′-cyclic phosphate termini. Accordingly, these characteristic ends are recognized by the players involved in circularization of the unit-lengths linear RNAs. For example, members of the *Pospiviroidae* family rely on a host RNA ligase that catalyzes ligation of 5′-phosphate and 3′-OH termini. The termini are brought into close proximity by a conformational switch upon cleavage of the long replication intermediates into unit-length strands (53).

In the family of *Avsunviroidae*, unit-length RNAs are formed by HHR mediated self-cleavage and thus contain 5′-OH and 2′, 3′-cyclic phosphate termini. The two termini are brought in the proper orientation by a conformational rearrangement (54) and are presumably circularized by a tRNA ligase (55). Alternatively, it has been suggested that circularization may occur by HHR catalysis producing a typical 3′, 5′-phosphodiester bond or by another type of self-ligation resulting in an atypical 2′, 5′-phosphodiester bond (56). A convincing candidate to perform self-ligation in three satellite RNAs of nepoviruses is the HPR. The ligase activity of the HPR *in vitro* is significantly higher than that of the HHR, and therefore it would be plausible that circularization *in vivo* is supported by the HPR alone (57,58). Self-cleavage of the human HDV ribozyme produces 5′-OH and 2′, 3′-cyclic phosphate termini in analogy to the mechanism used by the HHR and HPR. Early results suggested that the HDV ribozyme performs self-ligation also *in vivo*. However, more recent studies support circularization catalysis by host enzymes, presumably also by a tRNA ligase (59).

The functional advantage of all these circular viroids, viroid-like satellite RNAs and the HDV RNA is certainly protection against exonucleases, but also full-genome replication without the need for initiation or termination tags, something that obviously is relevant for primitive life forms. Furthermore, the HDV RNA encodes in its antigenomic strand, the delta antigene, demonstrating a clear functional link. This is not realized for the genomes of viroids and viroid-like satellite RNAs, which are too small to encode a protein of minimal complexity.

## CIRCULARIZATION *IN VITRO*

The broad occurrence of circRNAs *in vivo* and the study of their structural and functional properties have caused demand for methods that allow efficient preparation of circular RNAs *in vitro*. There is a choice of chemical and enzymatic protocols available, the majority however being more suitable to small and middle-size RNAs. Those in most cases can be chemically synthesized ensuring homogenous 5′- and 3′-ends. *In vitro* transcription is often associated with terminal heterogeneity, dramatically decreasing the yield of ligation for circularization. This can be overcome by the use of special protocols (60). However, circularization of larger RNAs remains challenging. Here, in particular, modified group I introns with permuted introns and exons (PIE strategy) have shown great promise. In general, chemical or enzymatic splint ligation strategies are more easily adaptable to any sequence of interest, whereas PIE and related RNA catalyzed methods suffer from sequence limitations. Proper folding of the RNA along with sequence requirements at the active site of the RNA catalysts are serious restrictions associated with these procedures. Nevertheless, there are also impressive examples of the preparation of large circular RNA molecules.

### Chemical ligation/synthesis

**Figure 4.**
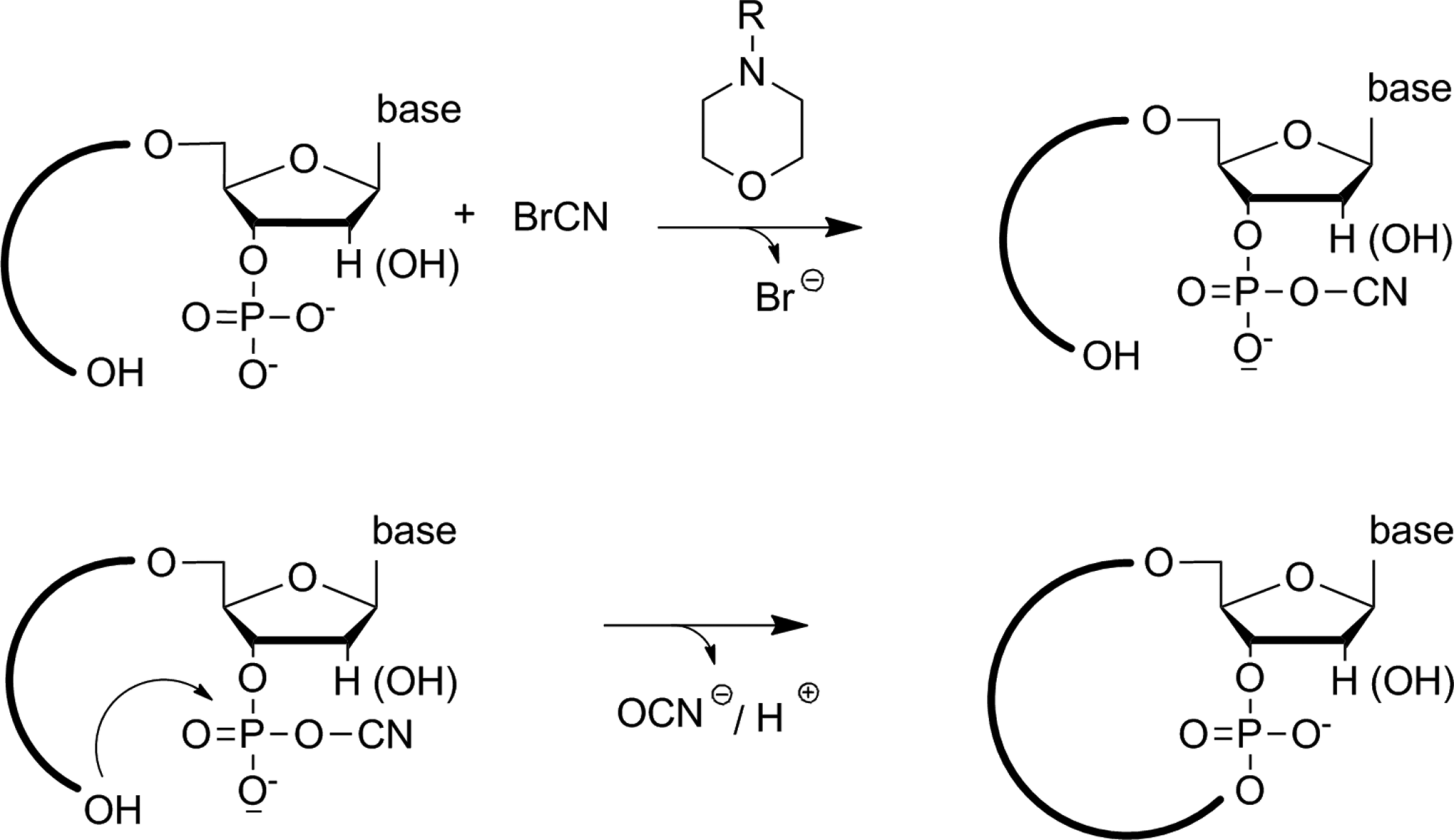
RNA circularization by chemical ligation with cyanogen bromide in the presence of a morpholino derivative as activator. R = CH_3_; CH_2_CH_2_SO_3_H.

Chemical ligation of nucleic acid strands was pioneered by Shabarova *et al*. using cyanogen bromide (BrCN) together with morpholino derivatives such as 2-(N-morpholino)-ethane sulfonic acid (MES) for linking two DNA strands carrying terminal 5′-hydroxyl and 3′-phosphate groups (61) (compare Figure 4).

**Figure 5.**
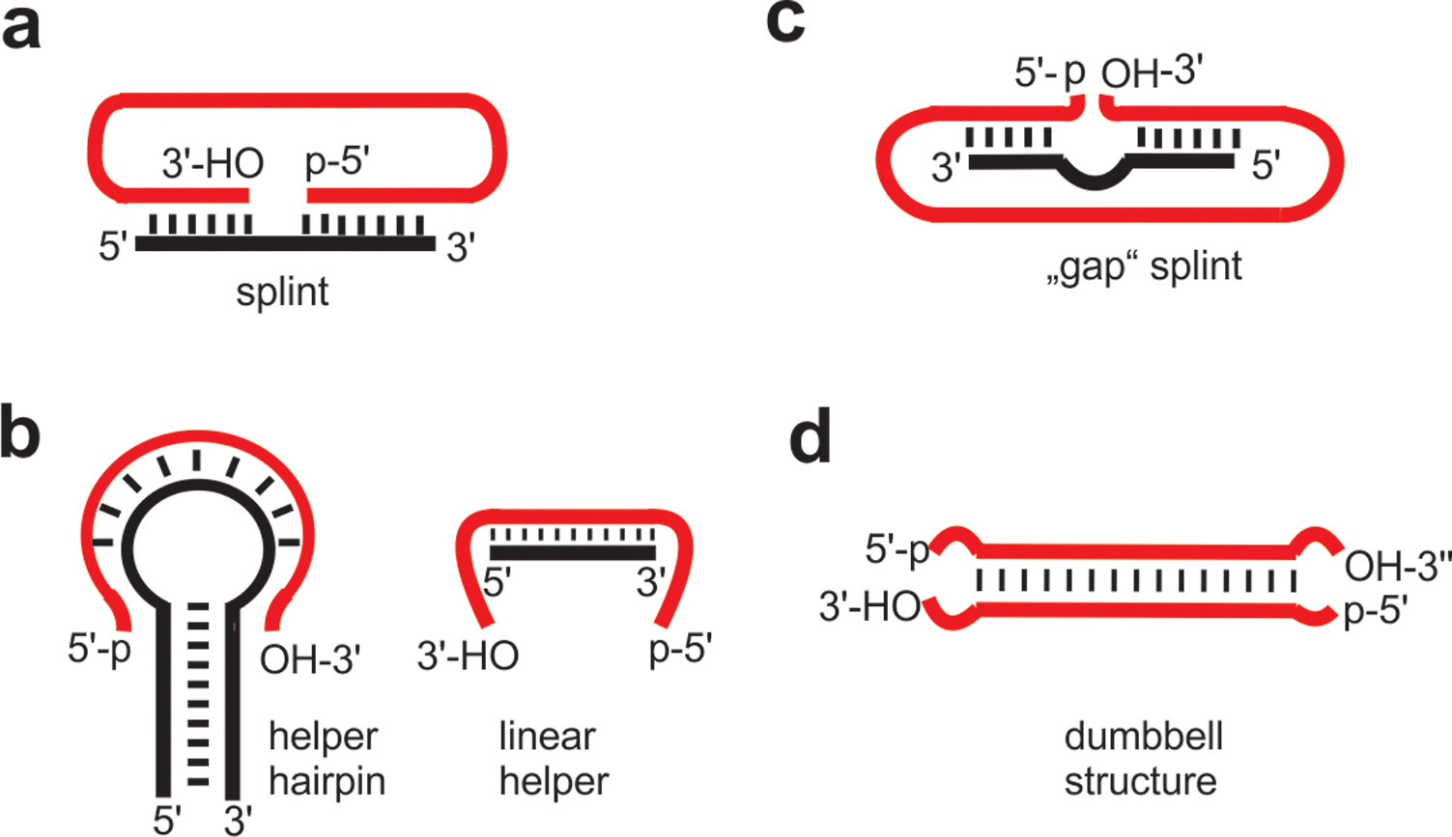
Strategies for juxtaposing reactive ends for enzymatic ligation. Pre-orientation of 5′-p and 3′-OH of the RNA substrate is achieved (**a**) by hybridization to a splint. This strategy can be used for ligation with T4 DNA ligase (DNA splint required), T4 RNA ligase 1 (RNA splint required) and T4 RNA ligase 2 (DNA or RNA splint) (68); (**b**) by hairpin and linear helper oligonucleotides (87); (**c**) by a gap splint that upon hybridization leaves the terminal two or three nucleotides of both ends single stranded at the ligation junction (88); (**d**) by favorable intrinsic structures (i.e. dumbbell folds (17)). Strategies (b), (d) and (c) are favorable for ligation with T4 RNA ligase 1. All strategies may be used also for chemical RNA ligation. Note that in this case a 3′-phosphate and a 5′-OH group are favorable.

Later on, other condensing agents such as ethyl-3-(3′-dimethylaminopropyl) carbodiimide (EDC) were used to support phosphodiester bond formation; however, BrCN appears to be the more widely used reagent (62,63). Chemical ligation strategies are also useful for circularization experiments (64,65) (Figure 4), provided that the two ends of a linear nucleic acid strand are brought in close proximity (compare Figure 5). This is typically achieved by an oligonucleotide splint that interacts with the two termini and thus allows circularization. Naturally, intermolecular ligation is a severe competition reaction, which however can be suppressed by working with small concentrations of the nucleic acid to be circularized. An even more serious side reaction associated with chemical ligation of RNA strands compared with DNA is the formation of 2′, 5′-phosphodiester bonds instead of the natural 3′, 5′-phosphodiester. In order to circumvent this problem, often oligonucleotides with a 2′-deoxy sugar moiety at the 3′-end of the oligonucleotide to be circularized are used (65,66) (Supplementary data, Protocols S1 and S2).

In 1999, Micura *et al*. reported an approach for solid phase synthesis of circular RNAs of 2–21 nucleotides length (67). The procedure combines standard phosphoramidite chemistry for chain assembly and phosphotriester chemistry for ring closure. A key element of the procedure is the start nucleoside being linked via a 3′-phosphodiester to the polymer support. After chain assembly is finished, final detritylation generates a 5′-terminal hydroxyl group for nucleophilic attack onto the unique 3′-terminal phosphodiester, leading to circularization while the oligonucleotide is still linked to the polymer. Subsequent cleavage of cyanoethyl groups enables selective detaching of circular RNAs. This is possible, because only the circRNAs are bound through phosphotriesters via an *o*-chlorophenyl group to the resin and therefore can be selectively removed by treatment with tetramethylguanidinium pyridine-2-aldoximate. Remaining non-circularized or mis-linked oligoribonucleotides are not removed due to their more stable phosphodiester linkages. This method allowed the production of circular RNAs with an average yield of 15% (67).

### Enzymatic ligation

A number of protocols for enzymatic ligation of synthetic oligonucleotides have been developed (68). Typically, three different polynucleotide ligases, capable of ligating nicks in single- and/or double-stranded RNA constructs are used: T4 DNA ligase (T4 Dnl), T4 RNA ligase 1 (T4 Rnl 1) and T4 RNA ligase 2 (T4 Rnl 2), all encoded in the genome of bacteriophage T4 (69,70). They catalyze the formation of a phosphodiester bond between 5′-phosphate (donor) and 3′-hydroxyl (acceptor) end groups in DNA or RNA in an ATP-dependent reaction.

Independent of the chemical or enzymatic preparation of the linear precursor, the 5′-terminus needs to be phosphorylated to provide the functionality required for ligation. Phosphorylation can be performed chemically or enzymatically (71) followed directly by the circularization reaction (72) (Supplementary data, Protocols S3 and S4). In the case of enzymatically prepared RNA substrates, the 5′-terminal triphosphate resulting from the *in vitro* transcription reaction must be removed prior to phosphorylation by a polynucleotide kinase. To circumvent the two enzymatic steps of de- and rephosphorylation, GMP-primed *in vitro* transcription by T7 RNA Polymerase is an elegant way for obtaining 5′-monophosphorylated linear RNA substrates (73,74), which can be directly used for RNA circularization (16,75). In the majority of publications, protocols for T4 DNA ligase as well as RNA ligase-mediated circularization of short RNAs (<500 nt) are described. Large substrates in kb scales require different strategies, due to structural challenges. Therefore, as mentioned above, for circularization of large RNAs, protocols using the PIE strategy (described below) may be the better alternative.

#### T4 DNA ligase

When using T4 DNA ligase, the ligation site needs to be located within a double-stranded region of the nucleic acid. Single-stranded regions are not ligated. An oligonucleotide splint can be used to create the required double-stranded region around the ligation site, and in addition to ensure juxtaposition of the 5′-phosphate and 3′-hydroxyl termini in duplex DNA or RNA (76) (Figure 5a). It is evident that temperature, stoichiometry and splint length have to be adjusted for each substrate. If the RNA is highly structured, longer splints up to full-length base pairing may be advantageous (77). However, this enhances the risk of unfavorable structures in the splint itself. The temperature optimum for the enzyme is 16°C; accordingly ligations are carried out preferentially at lower temperatures. T4 DNA ligase-mediated RNA ligation was pioneered by Moore and Sharp (78,79), and ever since has been used in a number of protocols. The method was shown suitable also for RNA circularization. A representative example is the circularization of a 453-nt long RNA that, in its circular form, was successfully translated *in vitro* (16) (Supplementary data, Protocol S5).

#### T4 RNA ligase 1

T4 RNA ligase 1 assists in the formation of 3′, 5′-phosphodiester bonds in single-stranded RNA molecules. Already in the early 1970s it has been shown that the enzyme can be used to produce a single-stranded circular product from a linear precursor with 3′-hydroxyl and 5′-phosphate termini (80). As mentioned above, intermolecular ligation is competing with ring formation. However, under suitable conditions, circularization can be made the predominant event. RNA chains with a minimal length of six to eight nucleotides could be circularized (81,82) (Supplementary data, Protocol S6). T4 RNA ligase 1 has different preferences for donor and acceptor nucleotides at the ligation site: A > G ≥ C > U for the 3′-terminal nucleotide acceptor, and pC > pU > pA > pG for the 5′-terminal nucleotide donor (83,84).

T4 RNA ligase 1 was used for circularization in a number of applications with short and long RNAs as substrates (for exemplary protocols see Supplementary data (Protocols S7 (81) and S8 (85)). An impressive example is the circularization of a linear RNA strand to yield an authentic infectious circular RNA of the *citrus exocortis* viroid strain A (CEV-A) (86). Furthermore, circular hammerhead ribozymes were synthesized from linear oligoribonucleotides using T4 RNA ligase *1* (87). The authors observed that circularization efficiency is strongly dependent on the nature of the RNA substrate; some of the linear RNAs could not be efficiently circularized under standard conditions. For those RNA substrates, helper deoxyoligonucleotides, preventing the RNA substrate from folding into unsuitable structures, were used (Figure 5b). In the presence of the helper strands, circular ribozymes as small as 15 nucleotides in length could be efficiently synthesized at concentrations as high as 50 μM in the ligation reaction. Linear and hairpin type helper oligonucleotides were used. In the presence of the linear helper oligonucleotide circRNA was generated with 40% yield, the hairpin helper allowed for ring formation with nearly 100% yield (87) (Protocol S9, Supplementary data). The circular products retained their biological activity; some displayed even increased catalytic activity and decreased need for divalent cations. In general, helper oligonucleotides have proven useful for proper orientation of the reactive ends. In addition to helper oligonucleotides that assist in ligation by interaction with the substrate in a distance from the ligation site (Figure 5b), also splints may be used that bring the reactive ends close together, but leave the terminal two to three nucleotides of both donor and acceptor single stranded. This is a common procedure for ssRNA ligation with T4 RNA ligase 1 (88,89), and should be a suitable strategy also for circularization (Figure 5c). Another scenario uses internal base pairing of the linear precursor to promote ligation (Figure 5d). Beaudry and Perreault developed a procedure for the synthesis of circular RNAs of any sequence based on internal secondary structures to position the 5′- and 3′-termini as single-stranded region, but held in close proximity for subsequent ligation (90). In another approach, dumb-bell-shaped RNAs were synthesized by closing the loops of linear but secondary folded precursors with T4 RNA ligase 1 (17,22,91–93) (for representative protocols see Supplementary data, Protocol S10).

#### T4 RNA ligase 2

Inter- and intramolecular ligation activity of T4 RNA ligase 2 was described by Ho and Shuman in 2002 (94). However, compared with its close relative T4 RNA ligase 1, this RNA ligase is much more efficient in joining nicks in dsRNA substrates, rather than connecting the ends of ssRNA (95,96). This characteristic feature was used to circularize a specific RNA substrate *in vitro* with the donor and acceptor ends hold in proximity by internal secondary structures (75) (see Supplementary data, Protocol S11). A detailed mechanistic and kinetic analysis of T4 RNA ligase 2 is described in (94). There is a homologous protein to T4 RNA ligase 2 occurring in vibriophage KVP40. It also catalyzes RNA circularization of single-stranded small (such as 18 mers) RNAs in an ATP-dependent manner (see (97) for detailed comparison and kinetic data of T4 and KVP40 RNA ligase 2-dependent circularization). Furthermore, a truncated version of T4 RNA ligase 2 with 249 amino acid residues is commercially available. It ligates the enzymatically or chemically pre-adenylated 5′-end of DNA (AppDNA) or RNA (AppRNA) to the 3′-end of RNA in the absence of ATP (94,98), and thus potentially can also be used for circularization.

#### Other RNA ligases with potential for in vitro RNA circularization

Apart from the three most widely used ligases described above, there are a number of other ligases capable of supporting RNA circularization. For example, tRNA ligases from wheat germ and a similar ‘ligase’ from *Saccharomyces cerevisae* may be applied for RNA ligation *in vitro* (99–101). Compared with RNA ligases 1 and 2, these ligases accept substrates with 5′-terminal phosphate donors and 2′-terminal phosphate acceptors (99,102). The formed internucleotide bond is a 2′-phosphomonoester, 3′, 5′-phosphodiester structure, as appearing in tRNA splicing intermediates *in vivo* (99,100). A recombinant yeast RNA ligase was shown to convert linear introns to their circular counterparts, although notably slower than wild-type *Arabidopsis* tRNA ligase (55).

Another RNA ligase was found in *Pyrococcus abyssi*, termed Pap1020. It performs the ATP-dependent circularization of oligoribonucleotides *in vitro*. Ring formation occurs by intramolecular reaction between the 3′- and 5′-termini of the RNA, whereby in contrast to T4 RNA ligase 1 or 2 no oligomerization was observed as side reaction (103). A wide range of temperatures from 20 to 95°C was screened and found suitable for efficient ligation. Varying the ATP concentration from 5 μM to 1 mM revealed that circularization activity decreases with increasing ATP concentration (see Supplementary data, Protocol S12).

Yet another ligase, named RtcB, was described by Tanaka and Shuman (104). It belongs to an *Escherichia coli* RNA repair operon with still unknown mechanistic features. Unlike classic ligases, RtcB seals broken RNAs with 3′-phosphate and 5′-OH termini. The reaction is dependent on GTP, which in the first step is bound to the protein via a histidinyl residue. The 3′-phosphate end takes over the guanylate and forms a polynucleotide-(3′)-pp-(5′)G intermediate. Subsequently, the 3′, 5′-phosphodiester bond is formed by nucleophilic attack of the 5′-terminal hydroxyl group onto the activated 3′-phosphate of the intermediate (105). The authors tested several GTP concentrations for RNA ligation and circularization, finding increasing yields of circRNA with increasing GTP concentration, up to complete ligation at 6.25 μM GTP (see Protocol S13, Supplementary data).

### Artificial rolling circle replication and HPR supported circularization

As described above, viroids and other small infectious RNAs replicate via the double rolling circle mechanism (1,50–51). An application using the rolling circle reaction and the self-splicing activity of the HPR for the generation of circular RNA was described by Kool and Diegelman (106). Circular single-stranded DNA templates were used for *in vitro* transcription by either T7 or *E. coli* RNA polymerase resulting in a multimeric repeating RNA sequence harboring the HPR element. Thus, upon transcription, RNA cleaved itself in monomer-length segments. The authors observed the subsequent formation of circular monomers and suggested that circularization occurs because of the intrinsic HPR activity (107). Data from our laboratory (75) and others (108,109) further support the idea of HPR-derived RNA circularization. We have demonstrated that specifically designed linear RNAs harboring the HPR structure undergo self-processing and circularization (75). Dallas *et al*. demonstrated that freezing stimulates the self-ligation (circularization) of linear forms of the HPR containing 2′,3′-cyclic phosphate and 5′-OH termini (108). In a similar study, self-processing of the 5′- and 3′-ends of a transcribed HPR derived precursor RNA followed by ligation of the processed ends to produce a circular RNA was shown (109).

### PIE method (RNA cyclase ribozyme)

**Figure 6.**
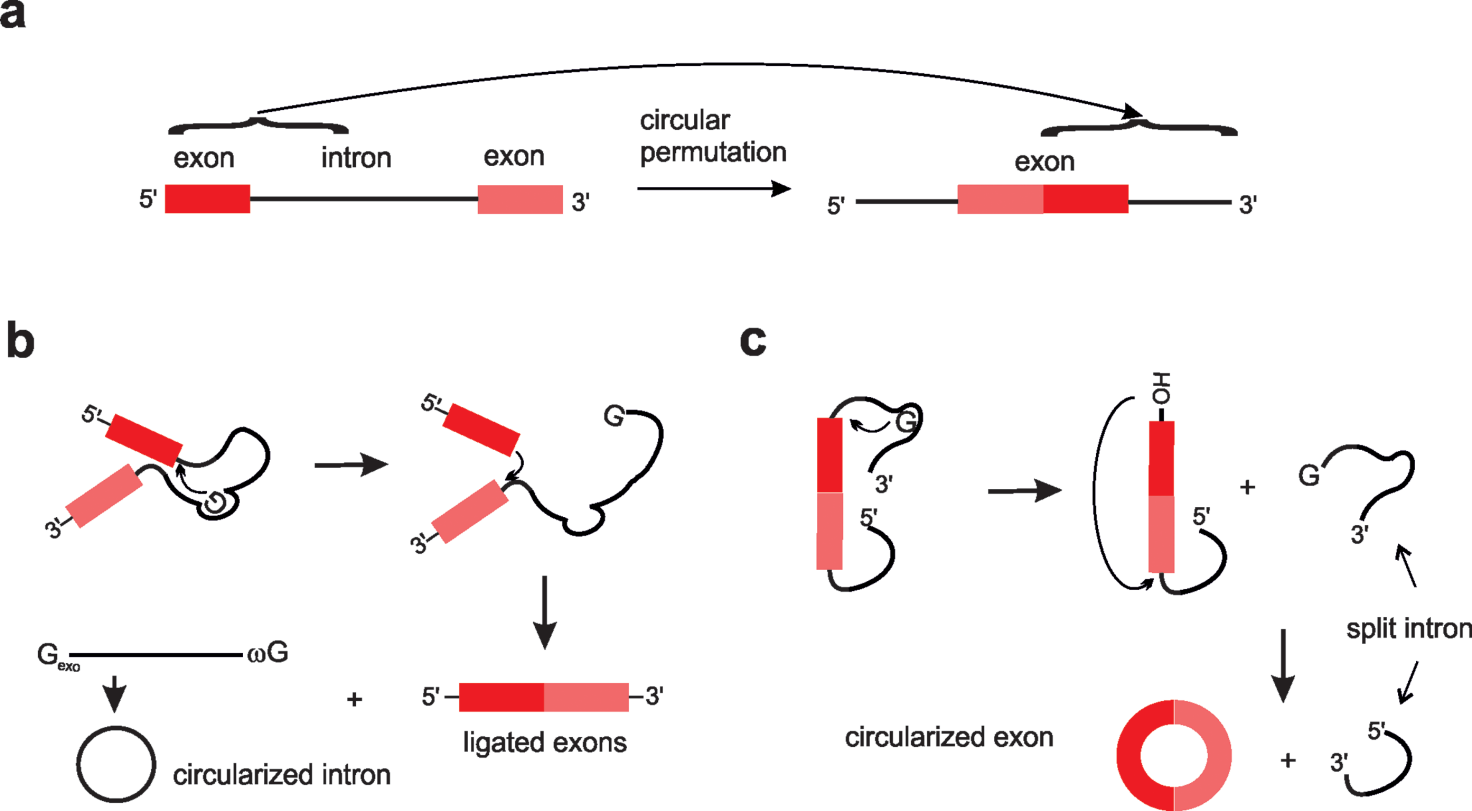
Comparison of group I intron self-splicing with the permuted intron exon method. (**a**) Illustration of the circular permutation of the intron. (**b**) Upon splicing in the normal intron, exons are ligated and the intron is circularized. (**c**) In the permuted intron, exons are circularized and split introns appear.

In contrast to the methods of chemical and enzymatic ligation, which can be merely applied *in vitro*, a spontaneous group I intron self-splicing system, designated as the PIE method, allows circular RNA production *in vitro* and *in vivo* (Figure 6). In 1992, Puttaraju and Been first reported that circularly permuted group I intron precursor RNAs derived from *Tetrahymena* or *Anabena*, containing end-to-end fused exons that interrupt half intron sequences (comp. Figure 6a), self-splice to generate a circular RNA exon *in vitro* (110). Ford and Ares Jr extended this work by demonstrating that foreign sequences can be placed in the exon of a permuted group I intron self-splicing system (here termed ‘cyclase ribozyme’) derived from the phage T4 *td* gene and made circular *in vitro* (111). This is possible, because the exon sequence does not participate in the self-splicing reaction. Expression of such constructs in *E. coli* and yeast resulted in the accumulation of circular RNAs (111). Mechanistically, the PIE method includes two transesterifications at defined splice sites as occurring in the normal group I intron self-splicing reaction. However, after splicing in the normal intron exons are ligated, whereas in the PIE are circularized (Figure 6b). The first transesterification leads to release of the 3′-terminal sequence (5′-half intron) of the PIE construct. The newly generated free 3′-OH group of the 3′-half exon attacks the 3′-splice site in the second transesterification. This results in circRNA and release of the 3′-half intron (Figure 6c).

Based on the PIE method, a wide variety of circular RNAs were generated. For example, circular RNA aptamers were produced *in vitro* and *in vivo* (21,112–114) as well as circular hammerhead ribozymes (115) and HDV ribozymes (116,117). Basically, it is possible to create any desired circular RNA by inserting the sequence into a particular site of a plasmid, creating a new RNA cyclase ribozyme gene. Circular RNAs in the range of 71–1130 nt were generated (118).

With the studies cited above, production of RNA circles by the PIE method in *E. coli* as well as in yeast was experimentally verified. However, so far the strategy has not been shown to work in mammalian cells.

### Modified group II introns

It is also possible to modify group II introns for inverse splicing *in vitro* generating RNA circles (119,120). Mikheeva *et al*. engineered a derivative of a yeast self-splicing group II intron that catalyzes the formation of a circular human exon *in vitro* (119). RNA circles were formed by inverse splicing, which requires arranging the exons consecutively, positioning the branch point upstream and intronic sequences up- and downstream of the exons. This design allows exon circularization upon two transesterifications, excluding any intronic sequences in the circle. An advantage of this strategy in comparison to group I intron mediated exon circularization is the somewhat higher efficiency and the complete sequence variability (for group I intron mediated exon circularization the 3′-terminal residue of the 5′-exon must be U). However, based on the group II intron self-splicing mechanism, circRNAs produced via this pathway carry a 2′,5′-phosphodiester at the ligation/circularization site.

## SUMMARY

Circular RNAs occur in all life-forms, eukaryotes, bacteria and archaea, as well as in proto-life forms like viroids, viroid-like satellite RNAs and (helper) viruses. Thus, evolution itself demonstrates the natural importance of circular RNAs. CircRNAs appear as intermediates and/or final product of RNA processing pathways and their occurrence appears to be ultimately linked to function (11,121–122). Being more stable than their linear counterparts, circular RNAs are likely candidates for storage of genetic information in early life forms, today still represented in viroids and virus-like particles. Accordingly, circRNA recently was discussed as a possible cyclic chromosome in a proto-cell referring to RNA world scenarios (123). RNA circularization once might have occurred randomly by the intrinsic catalytic activity contained in some RNAs. In modern life, ligation *in vivo* is mediated by proteinaceous ligases, but in some cases presumably also by still existing ribozyme activity.

Encouraged by the discovery of more and more circular RNA species *in vivo*, there has been growing interest in strategies for RNA circularization *in vitro*. Suitable models are needed for studying the structural and functional features of circular RNA occurring *in vivo*. Furthermore, applications in molecular biology, diagnostics or medicine will profit from the higher stability of circRNAs compared to their linear counterparts.

Dependent on the size and nature of the circular RNA to be prepared, the appropriate circularization method may be chosen. For example, the PIE method or group II intron inverse splicing can generate circRNAs *in vivo*; however, it does not allow for insertion of modified bases and/or sugar-phosphate backbones. *In vitro* production of circRNAs can satisfy this aim: modified nucleotides can be incorporated in RNA fragments by chemical synthesis, followed by chemical or enzymatic ligation/circularization. In general, the choice of the method should be based on three key points: the size of the circRNA, its nature (natural or modified, ss or dsRNA) and the site of production (*in vitro* or *in vivo*). Taken together, there are circularization strategies for all kinds of RNAs, and one may anticipate that the toolbox is still growing.

## SUPPLEMENTARY DATA

Supplementary Data are available at NAR Online.

SUPPLEMENTARY DATA
