# Supplementary material for: RNA circularization strategies in vivo and in vitro
Source: Nucleic Acids Res. 2015 Feb 6;43(4):2454–65. doi: 10.1093/nar/gkv045 (PMC4344496; doi:10.1093/nar/gkv045)
Supplement: SUPPLEMENTARY DATA [file supp_43_4_2454__index.html]

RNA circularization strategies in vivo and in vitro — RNA circularization strategies in vivo and in vitro — SUPPLEMENTARY DATA 

# RNA circularization strategies *in vivo* and *in vitro*

## SUPPLEMENTARY DATA

**Files in this Data Supplement:**

- SUPPLEMENTARY DATA
