# Supplementary material for: RNA circularization strategies in vivo and in vitro
Source: Nucleic Acids Res. 2015 Feb 6;43(4):2454–65. doi: 10.1093/nar/gkv045 (PMC4344496; doi:10.1093/nar/gkv045)
Supplement: SUPPLEMENTARY DATA [file supp_gkv045_nar-00828-survey-d-2014-File008.pdf]

Supplementary Data

RNA circularization strategies *in vivo* and *in vitro*

Sonja Petkovic and Sabine Müller

This PDF file includes:

Supplementary Protocols S1 to S13 p2-p7

References p8

Protocols

S1: RNA lariat production using cyanogen bromide

Nicked branched RNAs	Each 100 µM
DNA templates	Each 100 µM
MES	0.25 M
$MgCl_2$	20 mM
BrCN	1:10 vol of BrCN; 5 M CH_3CN

RNAs and DNAs were solved in MES. After addition of $MgCl_2$, samples were denatured at 95 °C for 10 min, slowly cooled down to room temperature, and then left at 4 °C overnight. The mixture was hold on ice for 15 min, then BrCN was added and the following reaction took place on ice within 15 min. Ligation was stopped by precipitation with 1 ml 2% $LiClO_4$ in acetone (1).

S2: Chemical circularization of RNAs with deoxyuridine at the 3'-end

pre-circRNA	50 µM
aligning DNA	55 µM
Imidazol-HCl (pH = 7.0)	200 mM
$NiCl_2$	100 mM
BrCN (solid)	to final concentration of 125 mM

Circularization was conducted for 12 h (2).

S3: Circularization of *in vitro* transcribed single stranded RNA

a) Dephosphorylation:

5'-triphosphate carrying RNA	100 ng
Tris-HCl	10 mM (pH 8.3)
$MgCl_2$	1 mM
$ZnCl_2$	1 mM
calf intestinal phosphatase	1 unit

Reaction took place at 37°C for 30 min, and was stopped by phenol extraction followed by ethanol precipitation.

b) Kinase reaction:

dephosphorylated RNA	20 µl
Tris-HCl	50 mM (pH 7.6)
$MgCl_2$	10 mM
ATP	100 µM
DTT	10 mM
polynucleotide kinase	5 units

Reaction proceeded at 37 °C for 30 min, and was stopped by heating to 65°C for 10 min.

c) Circularization:

RNA	2-3 µg/ml
Hepes	50 mM (pH 8.3)
$MgCl_2$	10 mM
ATP	1 mM
DTT	10 mM
DMSO	10%
T4 RNA ligase 1	100units/ml

Circularization took place at 12 °C over 4 h (3).

S4: Circularization of branched single-stranded RNA with T4 RNA ligase 1:

5'-phosphorylated RNA	1 µM
HEPES	3,75 mM (pH=7.5)
NaCl	15 mM
EDTA	0.1 mM
+ HEPES to final concentration of	5 mM
ATP	50 µM
$MgCl_2$	10 mM
DTT	10 mM
T4 RNA ligase 1	1 U/µL

RNA, HEPES, NaCl and EDTA was incubated and boiled at 95 °C for 3 min, followed by chilling on ice for 5 min. HEPES concentration was increased to 5 mM and ATP, $MgCl_2$, DTT and ligase were added as stated. Circularization was conducted at 37 °C for 30 min and stopped using 80 % formamide in 1x TBE, 50 mM EDTA and 0.025% each bromphenol blue and xyclene cyanol (4).

S5: Splinted RNA ligation using T4 DNA ligase

Plasmid DNAs were linearized with SmaI, followed by GMP primed *in vitro* transcription with T7 RNA polymerase [10:1 ratio of GMP to GTP]. RNA transcripts and DNA splint were mixed together and denaturated at 90 °C. Annealing took place by slow cooling to room temperature in a solution containing the following components within 60 min:

Tris-HCl, pH 7.5	10 mM
NaCl	100 mM
EDTA	0.1 mM
ATP	5 µM
T4 DNA ligase	100 units/ng transcript

Ligation buffer and T4 DNA ligase were added. Circularization proceeded at room temperature for 8 to 16 hours (5).

S6: One of the first circularization assays with T4 RNA Ligase 1

Tris-HCl, pH 7.5	50 mM
$MgCl_2$	10 mM
bovine serum albumine	20 pg/µl
ATP	0.1 mM
RNA [5'-^{32}P]-oligo(pA)	10^8 -10^9 cpm/µmol
T4 RNA ligase 1	0.005-0.02 U

Total reaction volume was 25 µl and circularization took place at 37 °C for 30 min. The mixture was then boiled for 2 min (6).

S7: RNA circularization with T4 RNA Ligase 1:

RNA [5'-^{32}P]- polyA (0.2 nmol of 5'-^{32}P-termini)	7 nmol
Tris-HCl, pH 7.5	5 µM
$MgCl_2$	1 µM
Albumin	5 µg
ATP	10 nmol
DTT	0.13 µmol
T4 RNA ligase 1	0.005-0.02 U

Total reaction volume was 100 µl and circularization took place at 38 °C for 30 min. Reaction was stopped by boiling for 2 min (6).

S8: Circularization of single-stranded oligoribo- or oligodeoxyribonucleotides with T4 RNA ligase 1:

RNA or DNA with 3'-hydroxyl terminus	6 pmol
Tris-HCl, pH 7.8	50 mM
$MgCl_2$	10 mM
DTT	10 mM
ATP	1 mM
hexamine cobalt chloride	1 mM
PEG 8000	25% (w/v)
T4 RNA ligase 1	20 U

Reaction was carried out for 16 hr at 22 °C in a total reaction volume of 10 µl and stopped by addition of 40 µl of 10 mM Tris-HCl, pH 8.0; 2.5 mM EDTA (7).

S9: Circularization of linear hammerhead ribozymes with T4 RNA ligase 1

RNA	0.03-100 µM
Helper DNA	0.036-120 µM
$MgCl_2$	10 mM
T4 RNA ligase 1	0.7 units/µL

RNA mixed with $MgCl_2$ was denatured (90 °C, 3 min) and incubated in T4 RNA ligase buffer at 16 °C for at least 1 h, before addition of T4 RNA ligase. Reaction was left to proceed at 16 °C overnight (8).

S10: Double-circularization of dumbbell shaped RNA with RNA ligase 1

5'-phosphorylated RNA	2 µM
ATP	1 mM
PEG 6000	25%
bovine serum albumin (BSA)	0.006%
Tris-HCl	50 mM (pH 7.5)
$MgCl_2$	10 mM
DTT	10 mM
T4 RNA ligase 1	0.05-0.4 units/µL

Reaction took place at room temperature overnight (9).

S11: Ligation of a nick in a double-stranded substrate with T4 RNA ligase 2:

dsRNA nicks with adjacent 3'-OH and 5'-p termini	10 to 20 pmol
Tris-HCl, pH 7.5	50 mM
$MgCl_2$	2 mM
DTT	1 mM
ATP	400 µM
T4 RNA ligase 2	1 U

Total reaction volume was 20 µl. Reaction proceeded for 30 min at 37 °C and was stopped by adding 2 µl of 0.5 M EDTA. (10)

S12: Exemplary protocol of RNA circularization using Pap1020:

labeled RNA substrate	1 pmol
Tris-HCl, pH 6.5	50 mM
$MgCl_2$	5 mM
DTT	5 mM
ATP	5 µM
Pap1020	2 pmol U

Incubate at 75 °C for 30 min (11).

S13: Exemplary protocol of RNA circularization with RtcB in a 10 µl reaction:

RNA substrate	0.1 µM
Tris-HCl, pH 8.0	50 mM
$MnCl_2$	2 mM
GTP	6.25 µM
Pap1020	1 µM

Incubation at 37 °C for 30 min. Reaction was stopped using 10 µl 90% (vol/vol) formamide and 50 mM EDTA (12)

References

1. Carriero, S. and Damha, M.J. (2003) Template-mediated synthesis of lariat RNA and DNA. *J Org Chem*, 68, 8328-8338.
2. Wang, S. and Kool, E.T. (1994) Circular RNA oligonucleotides. Synthesis, nucleic acid binding properties, and a comparison with circular DNAs. *Nucleic Acids Res*, 22, 2326-2333.
3. Rezaian, M.A. (1999) Synthesis of infectious viroids and other circular RNAs. *Curr Issues Mol Biol*, 1, 13-20.
4. Wang, Y. and Silverman, S.K. (2006) A general two-step strategy to synthesize lariat RNAs. *RNA*, 12, 313-321.
5. Chen, C.Y. and Sarnow, P. (1995) Initiation of protein synthesis by the eukaryotic translational apparatus on circular RNAs. *Science*, 268, 415-417.
6. Kaufmann, G., Klein, T. and Littauer, U.Z. (1974) T4 RNA ligase: substrate chain length requirements. *FEBS Lett*, 46, 271-275.
7. Nichols, N.M., Tabor, S. and McReynolds, L.A. (2008) RNA ligases. *Curr Protoc Mol Biol*, Chapter 3, Unit3 15.
8. Wang, L. and Ruffner, D.E. (1998) Oligoribonucleotide circularization by 'template-mediated' ligation with T4 RNA ligase: synthesis of circular hammerhead ribozymes. *Nucleic Acids Res*, 26, 2502-2504.
9. Abe, N., Abe, H., Nagai, C., Harada, M., Hatakeyama, H., Harashima, H., Ohshiro, T., Nishihara, M., Furukawa, K., Maeda, M. *et al.* (2011) Synthesis, structure, and biological activity of dumbbell-shaped nanocircular RNAs for RNA interference. *Bioconjug Chem*, 22, 2082-2092.
10. Nandakumar, J., Ho, C.K., Lima, C.D. and Shuman, S. (2004) RNA substrate specificity and structure-guided mutational analysis of bacteriophage T4 RNA ligase 2. *J Biol Chem*, 279, 31337-31347.
11. Brooks, M.A., Meslet-Cladiere, L., Graille, M., Kuhn, J., Blondeau, K., Myllykallio, H. and van Tilbeurgh, H. (2008) The structure of an archaeal homodimeric ligase which has RNA circularization activity. *Protein Sci*, 17, 1336-1345.
12. Chakravarty, A.K., Subbotin, R., Chait, B.T. and Shuman, S. (2012) RNA ligase RtcB splices 3'-phosphate and 5'-OH ends via covalent RtcB-(histidinyl)-GMP and polynucleotide-(3')pp(5')G intermediates. *Proc Natl Acad Sci U S A*, 109, 6072-6077.
